# Supplementary material for: Claudin-23 reshapes epithelial tight junction architecture to regulate barrier function
Source: Nat Commun. 2023 Oct 5;14:6214. doi: 10.1038/s41467-023-41999-9 (PMC10556055; doi:10.1038/s41467-023-41999-9)
Supplement: Supplementary file 1 — Supplementary Information [file 41467_2023_41999_MOESM1_ESM.pdf]

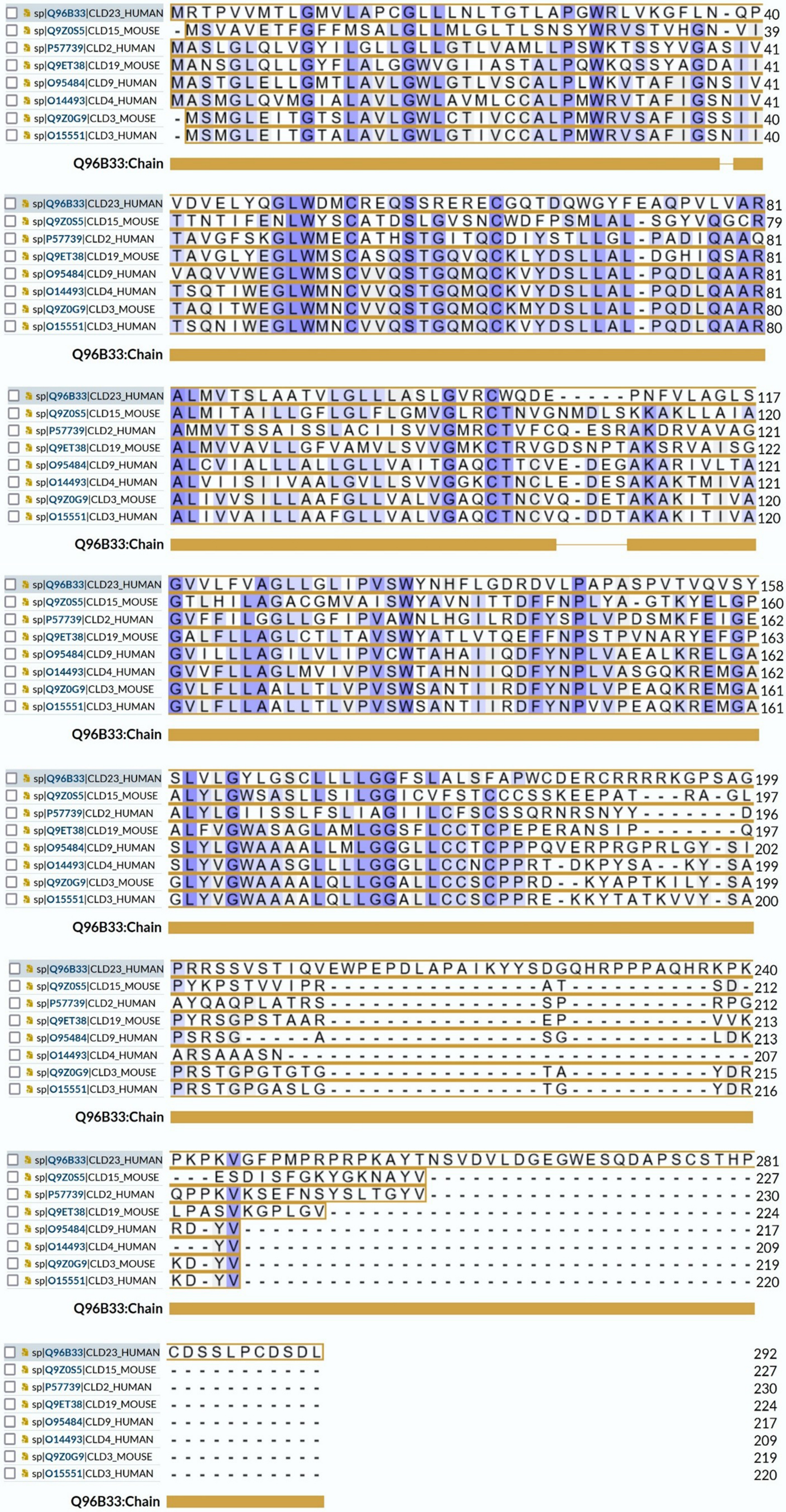

Supplementary Fig. 1: Multiple sequence alignment of CLDN23 and some classic and non-classical claudins. Sequence alignment of multiple CLDN family members with human CLDN23. The sequence alignment was determined using https://uniprot.org/align.

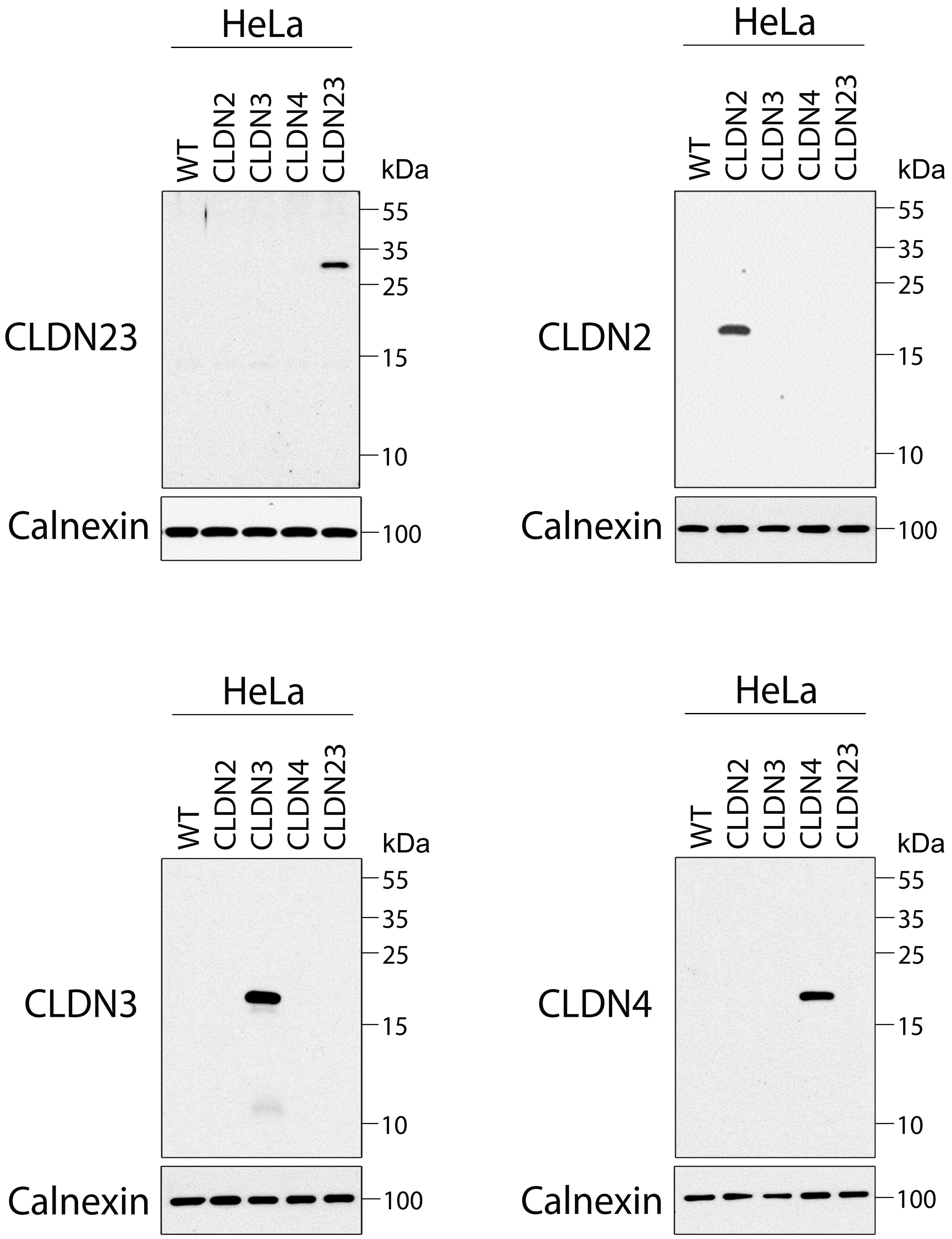

Supplementary Fig. 2: Specificity of a homemade anti-CLDN23 antibody. Specificity of the mouse antibodies against CLDN2, CLDN3, and CLDN4, and our in-house rabbit anti-CLDN23 antibody was tested using HeLa cells expressing each individual CLDN. Cell lysates were immunoblotted with the mouse antibodies against CLDN2, CLDN3, and CLDN4, as well as rabbit antibody against CLDN23. No cross-reactivity was detected between antibodies against CLDN2, CLDN3, CLDN4, and CLDN23 in lysates of HeLa cells expressing individual CLDNs. Calnexin was used as loading control. Immunoblot images are representative of two independent experiments.

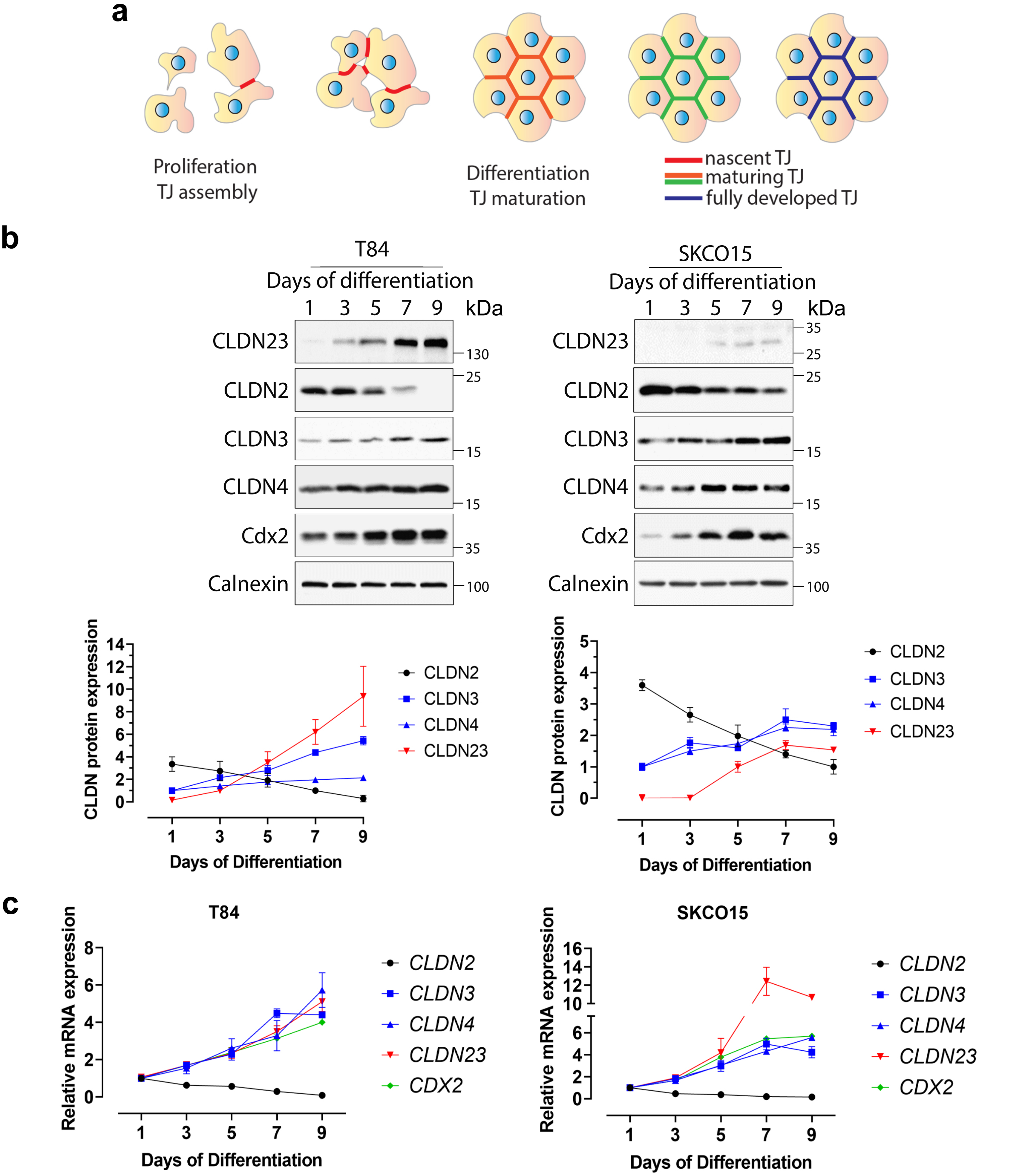

Supplementary Fig. 3: CLDN23 is upregulated during intestinal epithelial cell differentiation and TJ maturation. (a) Schematic of in vitro model of IEC differentiation and maturation of TJs. (b) Upper panel, representative Immunoblot image of CLDN23 protein expression in cell lysates from SKCO15 and T84 cells that formed confluent and differentiated for nine days in culture. CLDN23 up-regulation increased expression of barrier-forming CLDN3 and CLDN4 and a down-regulated channel-forming CLDN2 in both IEC lines. Lower panel shows densitometric analysis of the Immunoblots. Data are mean ± SD from three independent densitometric analyses. CDX2 and Calnexin were used as IEC differentiation marker and loading control respectively. (c) RTqPCR analysis of expression of *CLDN2*, *CLDN3*, *CLDN4*, and *CLDN23* as well as differentiation marker *CDX2* in both SKCO15 and T84 IEC monolayers allowed to differentiate for nine days in culture. The relative mRNA expression was calculated by the $2^{\Delta\Delta Ct}$ method and normalized to the housekeeping gene TATA box-binding protein (*TBP*). Results show the mean ± SD and are representative of two independent experiments, each one assayed in two technical replicates.

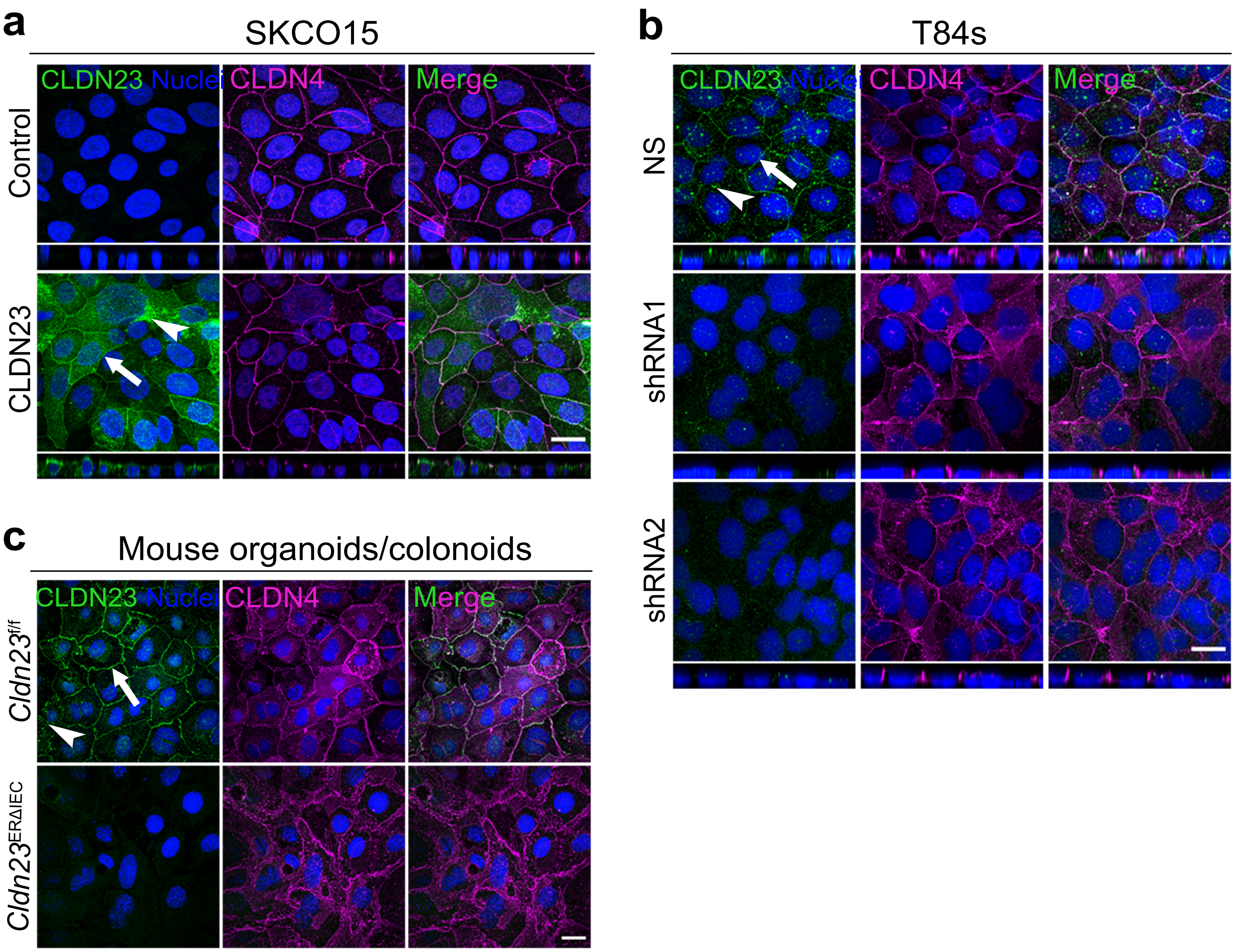

Supplementary Fig. 4: Intestinal epithelial cell lines with stable overexpression or silencing of CLDN23. Representative confocal images of either (a) SKCO15 control and CLDN23 overexpressing monolayers, (b) T84 control (NS) and KD (shRNA1 and shRNA2) IECs and (c) murine colonoid co-cultures derived from tamoxifen treated *Cldn23*$^{ER\Delta IEC}$ and *Cldn23*$^{f/f}$ mice stained with anti-CLDN23 (green) and anti-CLDN4 (magenta), with DAPI (blue) as a nuclear counterstain. Scale bar: 20μm. CLDN23 expression at cell-cell contacts and cytoplasm is indicated by arrows and arrowheads, respectively.

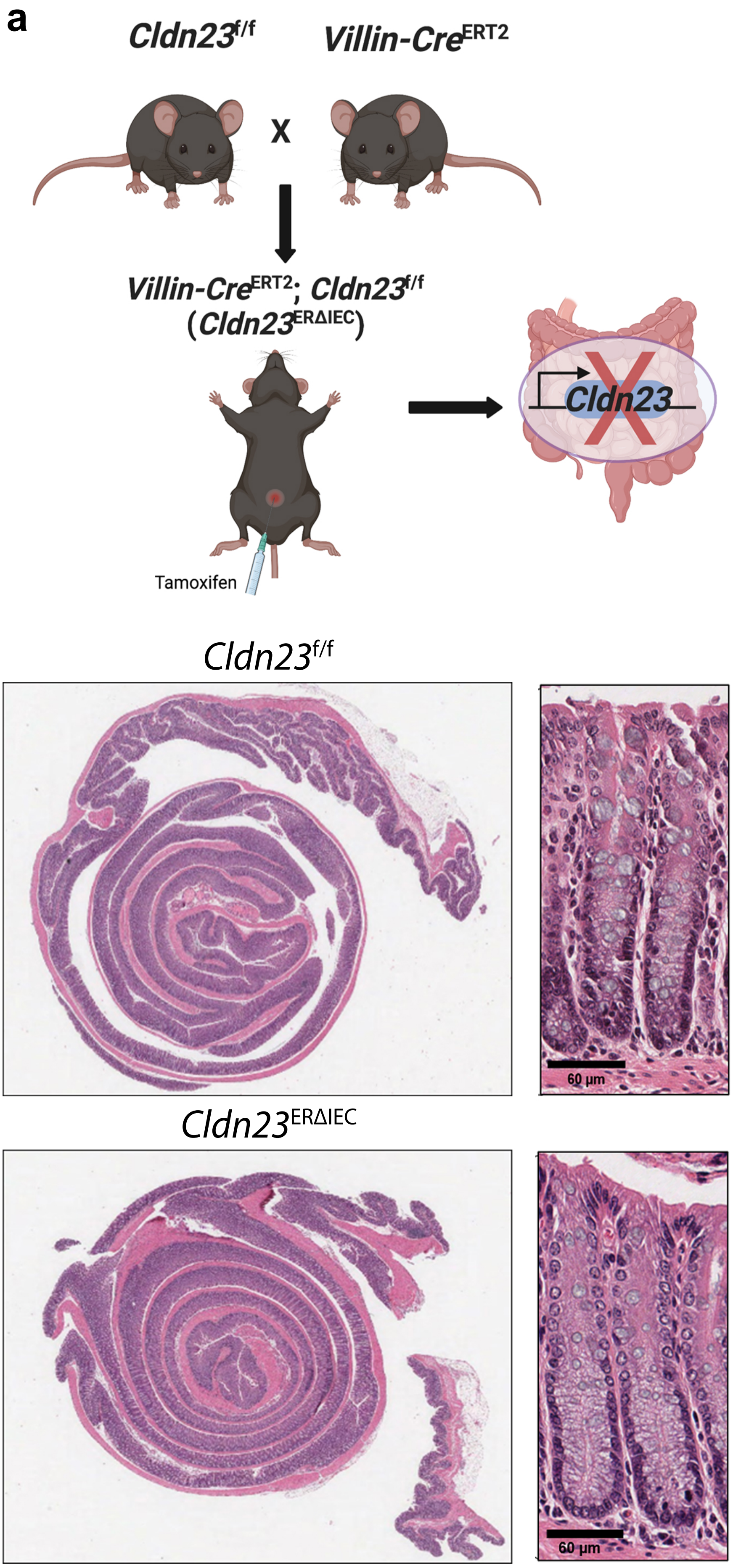

Supplementary Fig. 5: Loss of CLDN23 in IECs did not alter intestinal mucosal architecture. (a) Schematic of the generation of the IEC-specific Cldn23 knockout mice. *Cldn23*$^{f/f}$ mice were crossed with Villin-CreERT2 mice to generate *Cldn23*$^{ER\Delta IEC}$ mice. Cre+ mice were then injected with tamoxifen intraperitoneally for 5 days and rested for 3 weeks to attain deletion of CLDN23 in IECs. Schematic created with BioRender.com. (b) Hematoxylin-Eosin (H&E) staining of sections of Swiss roll mounts of the entire colon in *Cldn23*$^{ER\Delta IEC}$ and control littermates *Cldn23*$^{f/f}$ treated with tamoxifen for 5 days and analyzed 21 days after the last injection. Images are representative of two independent experiments. Scale bar is 60µm.

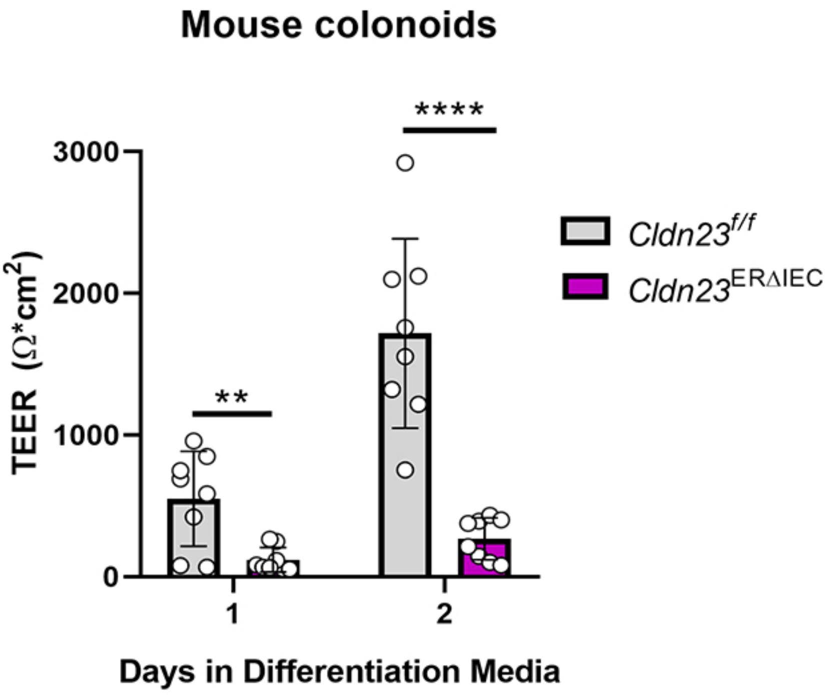

Supplementary Fig. 6: Loss of CLDN23 in colon-derived organoids results in reduced epithelial barrier function. TEER of colon-derived organoids from *Cldn23*$^{ER\Delta IEC}$ and control *Cldn23*$^{f/f}$ mice was measured for 2 days in monolayers cultured in differentiation media. Data are mean + SD and represent three independent experiments, each with at least two technical replicates. ***p≤0.001; two-way ANOVA with Tukey's posttest.

`

Percent Identity Matrix

	CLD23_Human	CLD15_Mouse	CLD2_Human	CLD19_Mouse	CLD9_Human	CLD4_Human	CLD3_Mouse	CLD3_Human
sp|Q96B33|CLD23_HUMAN	100.00%	22.97%	24.00%	25.23%	33.49%	28.43%	25.23%	24.65%
sp|Q9Z0S5|CLD15_MOUSE	22.97%	100.00%	27.35%	34.10%	34.62%	37.07%	35.07%	34.91%
sp|P57739|CLD2_HUMAN	24.00%	27.35%	100.00%	38.57%	38.86%	39.51%	37.56%	36.45%
sp|Q9ET38|CLD19_MOUSE	25.23%	34.10%	38.57%	100.00%	45.50%	42.93%	45.07%	44.86%
sp|O95484|CLD9_HUMAN	33.49%	34.62%	38.86%	45.50%	100.00%	64.56%	65.42%	65.12%
sp|O14493|CLD4_HUMAN	28.43%	37.07%	39.51%	42.93%	64.56%	100.00%	69.08%	69.71%
sp|Q9Z0G9|CLD3_MOUSE	25.23%	35.07%	37.56%	45.07%	65.42%	69.08%	100.00%	91.32%
sp|O15551|CLD3_HUMAN	24.65%	34.91%	36.45%	44.86%	65.12%	69.71%	91.32%	100.00%

Supplementary Fig. 7: Identity matrix for homology modeling of claudins studied in this work and the crystal structures used for modeling for their respective 3D structures. Identity matrix across modeled sequences and the respective crystal structures used to model CLDN2, CLDN3, CLDN4, and CLDN23 structures are highlighted with a green box. Claudin-23 has 33.49% identity with hClaudin-9 crystal structure. Claudin-2 – 39.5% identity with hClaudin-4 crystal structure. Claudin-3 – 91.3% identity with mClaudin-3 crystal structure. The identity matrix was determined using https://uniprot.org/align.

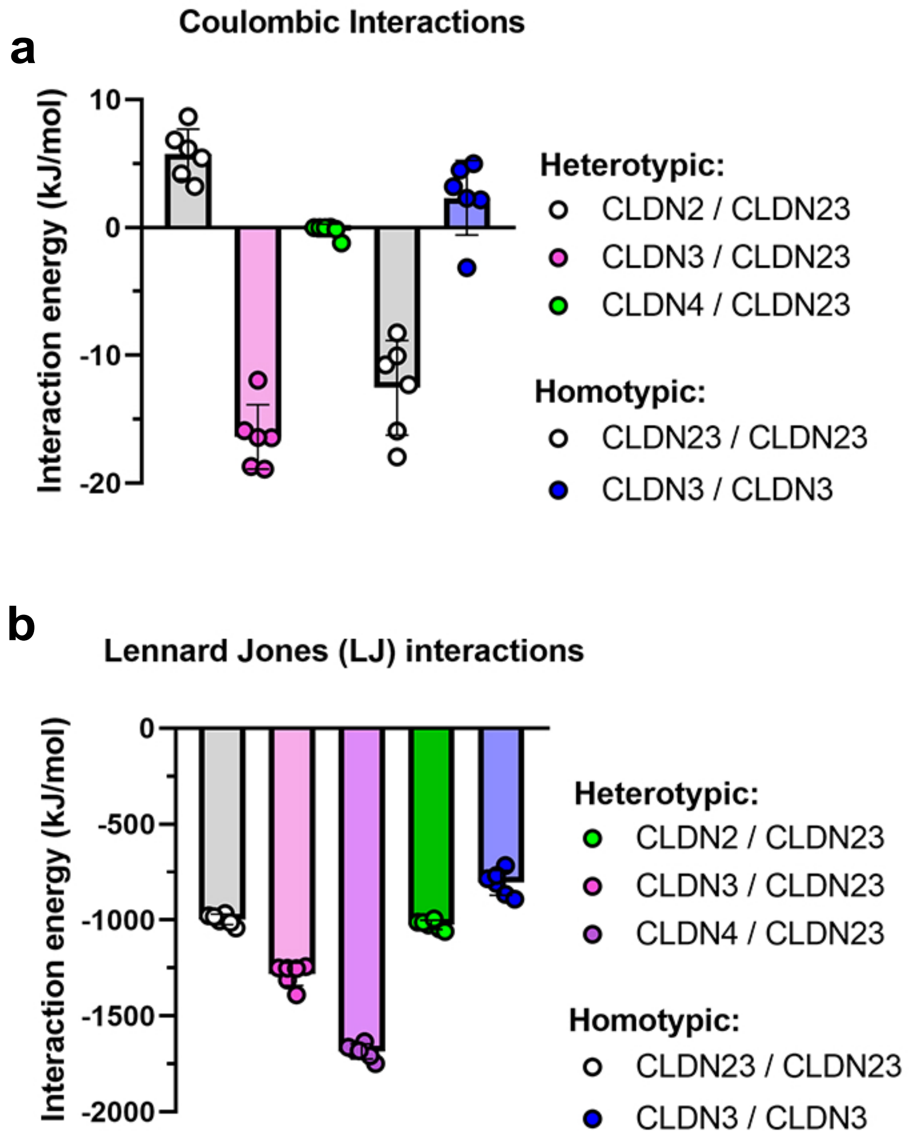

Supplementary Fig. 8: Coulombic and Lennard Jones interaction energies for heterotypic and homotypic CLDN pairs. The values were computed for the computationally relaxed structures shown in Figure 8 (e-h).

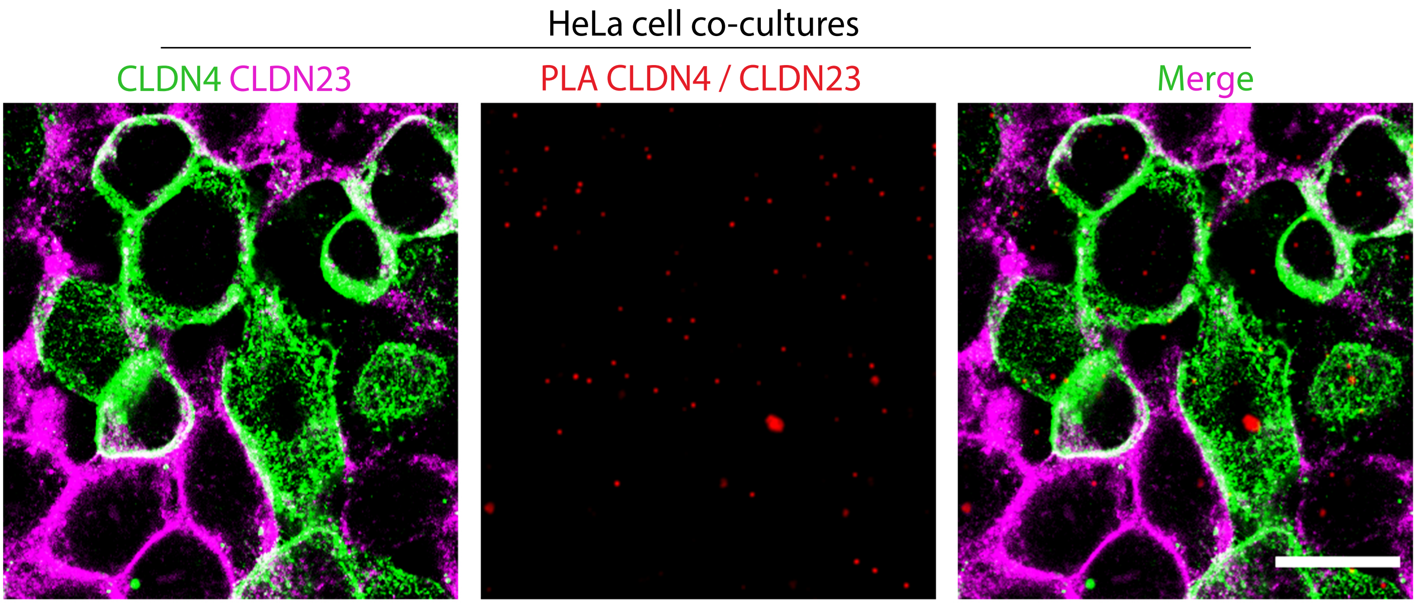

Supplementary Fig. 9: Trans interactions between CLDN23 and CLDN4 in HeLa cell co-cultures are not detected by proximity ligation assay. HeLa cells expressing CLDN4 (green) were co-cultured with HeLa cells expressing CLDN23 (magenta). The association in trans between CLDN4 and CLDN23 was investigated by in situ proximity ligation assay (PLA, red), employing a rabbit antibody against the cytosolic tail of CLDN23 and mouse antibody against the cytosolic tail of CLDN4. Scale bar is 20µm.

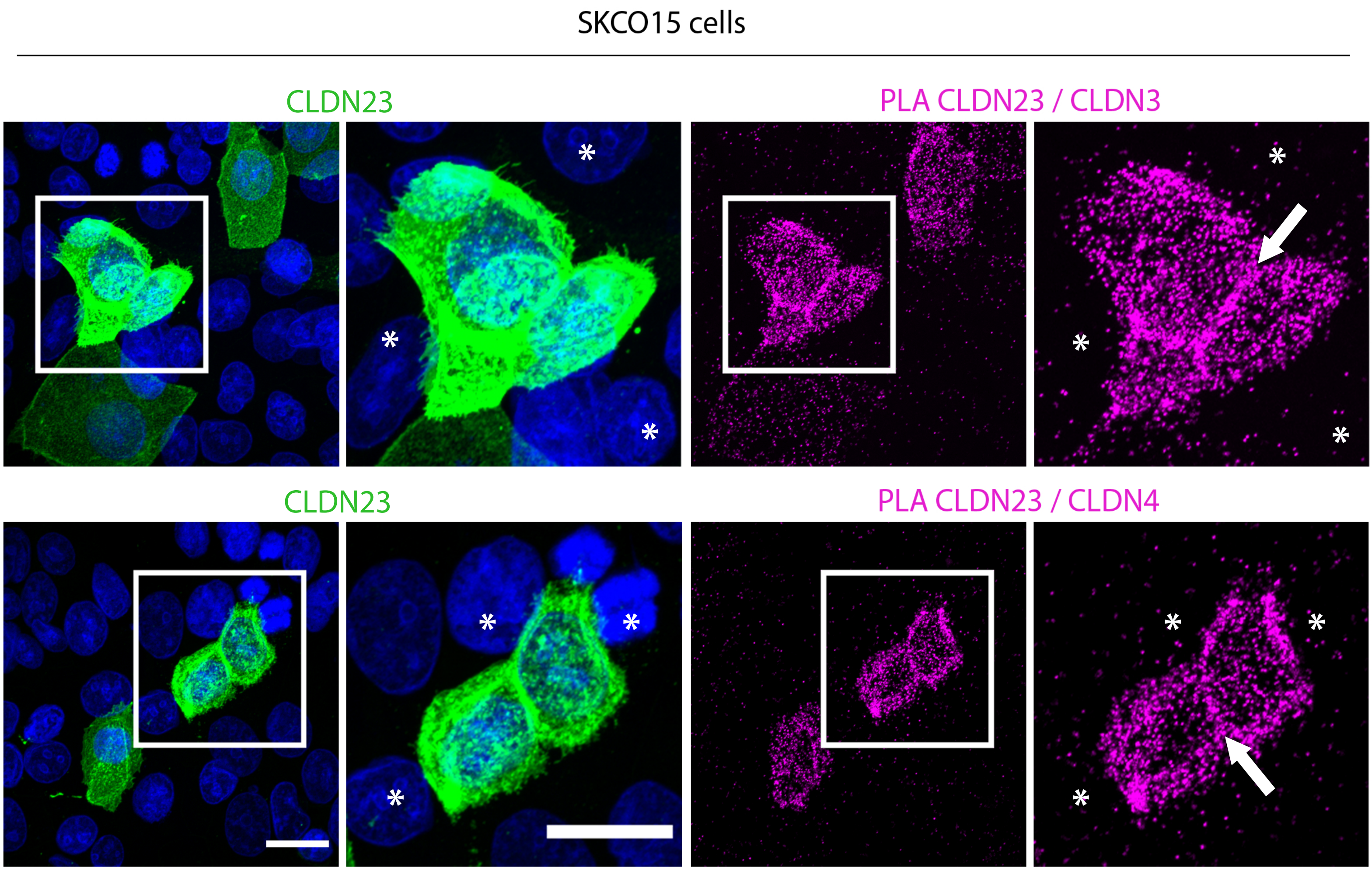

Supplementary Fig. 10: Specific association between CLDN23 and CLDN3 or CLDN4 is only detected in CLDN23-overexpressing SKCO15 cells. Representative confocal images show PLA signals at cell-cell contacts (arrow, magenta) with CLDN3 and CLDN4 in CLDN23-positive cells (green) in comparison to the surrounding un-transfected cells (asterisk). Scale bar: 20µm.

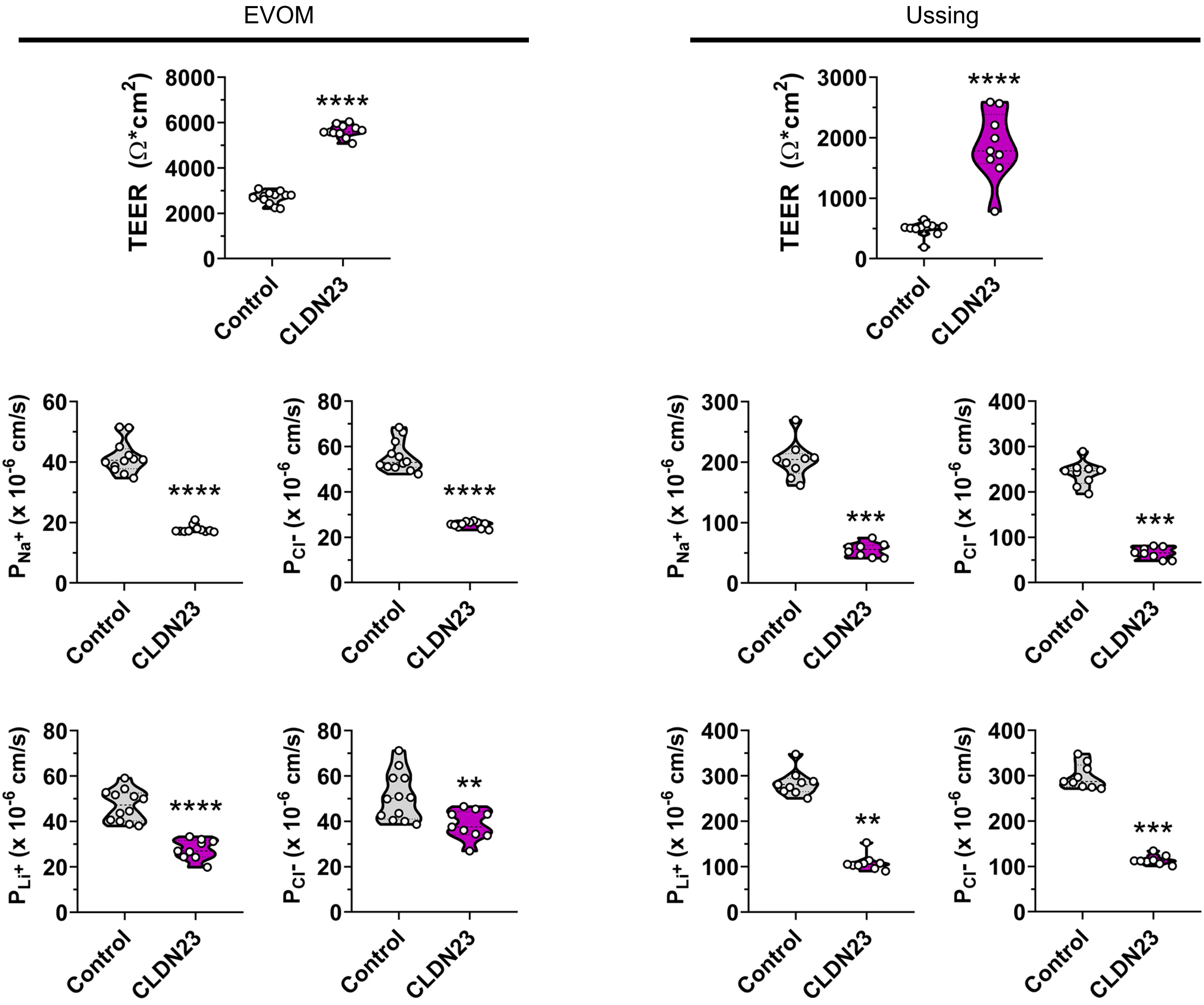

Supplementary Fig. 11. CLDN23 decreases paracellular permeability of Na^{+}, Li^{+} and Cl^{-} ions in SKCO15 cells overexpressing CLDN23. TEER and ion flux assays were performed in model epithelial SKCO15 control and CLDN23 overexpressing cells by EVOM epithelial voltohmeter (left) and Ussing chamber (right). Representative graphs of TEER and individual P_{Na+}, P_{Li+} and P_{Cl-} permeability in IEC monolayers cultured for 5 days. Data are mean + SD and represent one experiment, each with at least 10 technical replicates. $**p\leq 0.01$, $***p\leq0.001$, $****p\leq0.0001$; two-tailed Student's t test.
