# Supplementary material for: Claudin-23 reshapes epithelial tight junction architecture to regulate barrier function
Source: Nat Commun. 2023 Oct 5;14:6214. doi: 10.1038/s41467-023-41999-9 (PMC10556055; doi:10.1038/s41467-023-41999-9)
Supplement: Supplementary file 3 — Reporting Summary [file 41467_2023_41999_MOESM3_ESM.pdf]

nature portfolio

Corresponding author(s): Asma Nusrat, Shikha Nangia

Last updated by author(s): 2023/09/11

Reporting Summary

Nature Portfolio wishes to improve the reproducibility of the work that we publish. This form provides structure for consistency and transparency in reporting. For further information on Nature Portfolio policies, see our Editorial Policies and the Editorial Policy Checklist.

Statistics

For all statistical analyses, confirm that the following items are present in the figure legend, table legend, main text, or Methods section.

n/a	Confirmed	
☐	☒	The exact sample size (*n*) for each experimental group/condition, given as a discrete number and unit of measurement
☐	☒	A statement on whether measurements were taken from distinct samples or whether the same sample was measured repeatedly
☐	☒	The statistical test(s) used AND whether they are one- or two-sided<br>*Only common tests should be described solely by name; describe more complex techniques in the Methods section.*
☒	☐	A description of all covariates tested
☐	☒	A description of any assumptions or corrections, such as tests of normality and adjustment for multiple comparisons
☐	☒	A full description of the statistical parameters including central tendency (e.g. means) or other basic estimates (e.g. regression coefficient) AND variation (e.g. standard deviation) or associated estimates of uncertainty (e.g. confidence intervals)
☐	☒	For null hypothesis testing, the test statistic (e.g. *F*, *t*, *r*) with confidence intervals, effect sizes, degrees of freedom and *P* value noted<br>*Give P values as exact values whenever suitable.*
☒	☐	For Bayesian analysis, information on the choice of priors and Markov chain Monte Carlo settings
☒	☐	For hierarchical and complex designs, identification of the appropriate level for tests and full reporting of outcomes
☐	☒	Estimates of effect sizes (e.g. Cohen's *d*, Pearson's *r*), indicating how they were calculated

Our web collection on statistics for biologists contains articles on many of the points above.

Software and code

Policy information about availability of computer code

Data collection	N/A
Data analysis	N/A

For manuscripts utilizing custom algorithms or software that are central to the research but not yet described in published literature, software must be made available to editors and reviewers. We strongly encourage code deposition in a community repository (e.g. GitHub). See the Nature Portfolio guidelines for submitting code & software for further information.

Data

Policy information about availability of data

All manuscripts must include a data availability statement. This statement should provide the following information, where applicable:

- Accession codes, unique identifiers, or web links for publicly available datasets
- A description of any restrictions on data availability
- For clinical datasets or third party data, please ensure that the statement adheres to our policy

The source data, including original blots, immunofluorescence images, and quantification data are provided as a Source Data file. Source data are provided with this paper.

Research involving human participants, their data, or biological material

Policy information about studies with human participants or human data. See also policy information about sex, gender (identity/presentation), and sexual orientation and race, ethnicity and racism.

Reporting on sex and gender	This is not a clinical study. Discarded human material that is de-identified was used for determining expression of CLDN23 in the intestine. Thus, material from subjects is anonymous per US National Institute of Health and Institutional guidelines.
Reporting on race, ethnicity, or other socially relevant groupings	N/A
Population characteristics	N/A
Recruitment	N/A
Ethics oversight	N/A

Note that full information on the approval of the study protocol must also be provided in the manuscript.

Field-specific reporting

Please select the one below that is the best fit for your research. If you are not sure, read the appropriate sections before making your selection.

- [x] Life sciences
- [ ] Behavioural & social sciences
- [ ] Ecological, evolutionary & environmental sciences

For a reference copy of the document with all sections, see nature.com/documents/nr-reporting-summary-flat.pdf

Life sciences study design

All studies must disclose on these points even when the disclosure is negative.

Sample size	We used power analysis to determine sample size.
Data exclusions	Only few data points that were considered as outliers by the GraphPad 9 Prism program were excluded.
Replication	We confirm that all attempts at replication were successful. This is detailed in the figure legends of the manuscript.
Randomization	N/A. This is not a clinical study.
Blinding	We were blinded for in vivo data allocation and analysis.

Reporting for specific materials, systems and methods

We require information from authors about some types of materials, experimental systems and methods used in many studies. Here, indicate whether each material, system or method listed is relevant to your study. If you are not sure if a list item applies to your research, read the appropriate section before selecting a response.

Materials & experimental systems

n/a	Involved in the study	
[ ]	[x]	Antibodies
[ ]	[x]	Eukaryotic cell lines
[x]	[ ]	Palaeontology and archaeology
[ ]	[x]	Animals and other organisms
[x]	[ ]	Clinical data
[x]	[ ]	Dual use research of concern
[x]	[ ]	Plants

Methods

n/a	Involved in the study	
[x]	[ ]	ChIP-seq
[x]	[ ]	Flow cytometry
[x]	[ ]	MRI-based neuroimaging

Antibodies

Antibodies used	The following primary monoclonal and polyclonal antibodies were used to detect proteins by immunofluorescence (IF) or immunoblot (IB). From rabbit: anti-human/mouse CLDN23 (IB: 1/1,000; IF: 1/100) was generated; anti-human/mouse CLDN3 (Sigma, Cat. 218317, IB:1/1,000; IF:1/100); anti-human CDX2 (Cell Signaling, Cat. 39775, IB: 1/1000); and anti-calnexin (Cat. PA5-34665; IB: 1/20,000). From mouse: anti-mouse CLDN2 (Invitrogen, Cat. 32-5600; IB: 1/1000; IF: 1/250); anti-human CLDN3 (Sigma, Cat. SAB4200758, IB:1/1,000; IF:1/100); and anti-human CLDN4 (Invitrogen, Cat. 32-9400, IB:1/2000; IF:1:200); anti-human/mouse ZO-1 (Thermofisher, Cat. 33-9100, IB: 1:1000, IF 1:100).
Validation	All antibodies were validated for specificity by western blotting and immunofluorescence using cells with knockdown of proteins and their respective controls.

Eukaryotic cell lines

Policy information about cell lines and Sex and Gender in Research

Cell line source(s)	ATCC in USA
Authentication	Cell lines have been authenticated by the ATCC. One cell line, SKCO15 cells were provided and authenticated by Dr. Rodriguez-Boulan E (Le Bivic A., Real F.X., Rodriguez-Boulan E. Vectorial targeting of apical and basolateral plasma membrane proteins in a human adenocarcinoma epithelial cell line. Proc. Natl. Acad. Sci. U.S.A. 86:9313-9317(1989) (PubMed ID 2687880)).
Mycoplasma contamination	We perform regular testing for mycoplasma by PCR.
Commonly misidentified lines (See ICLAC register)	N/A

Animals and other research organisms

Policy information about studies involving animals; ARRIVE guidelines recommended for reporting animal research, and Sex and Gender in Research

Laboratory animals	Mice selectively deficient in CLDN23 in the intestinal epithelium were generated by breeding Cldn23 “floxed” (Cldn23f/f) mice with mice expressing the inducible mutated estrogen receptor fused to Cre-recombinase under control of the Villin promoter (Cldn23ERΔIEC). Six- to eight-week-old Cldn23ERΔIEC and control Cldn23f/f were injected intraperitoneally with 1mg/100µl of tamoxifen (Sigma, Cat. T5648) dissolved in 10% ethanol and sterile corn oil (Sigma, Cat. C8267) for 5 consecutive days. Animals were used 21 days after the last tamoxifen injection. Mice were kept under strict specific pathogen-free conditions with ad libitum access to normal chow and water. All mice were in C57BL/6J background.
Wild animals	None
Reporting on sex	Female and male mice were used indistinctly for all the experiments.
Field-collected samples	N/A
Ethics oversight	All experiments were approved and conducted in accordance with the guidelines set by the University of Michigan Institutional Animal Care and Use Committee.

Note that full information on the approval of the study protocol must also be provided in the manuscript.
